# Transcriptomic analysis of 3D Cardiac Differentiation of Human Induced Pluripotent Stem Cells Reveals Faster Cardiomyocyte Maturation Compared to 2D Culture

**DOI:** 10.1038/s41598-019-45047-9

**Published:** 2019-06-25

**Authors:** Mariana A. Branco, João P. Cotovio, Carlos A. V. Rodrigues, Sandra H. Vaz, Tiago G. Fernandes, Leonilde M. Moreira, Joaquim M. S. Cabral, Maria Margarida Diogo

**Affiliations:** 1 Department of Bioengineering and iBB-Institute for Bioengineering and Biosciences, Instituto Superior Técnico, Universidade de Lisboa, 1049-001 Lisbon, Portugal; 2 The Discoveries Centre for Regenerative and Precision Medicine, Lisbon Campus, Instituto Superior Técnico, Universidade de Lisboa, 1049-001 Lisbon, Portugal; 3 Instituto de Medicina Molecular, Faculdade de Medicina, Universidade de Lisboa, 1649-028 Lisbon, Portugal; 4 Instituto de Farmacologia e Neurociências, Faculdade de Medicina da Universidade de Lisboa, 1649-028 Lisbon, Portugal

**Keywords:** Differentiation, Stem-cell differentiation, Induced pluripotent stem cells, Stem-cell differentiation

## Abstract

Human induced pluripotent stem cells (hiPSCs) represent an almost limitless source of cells for disease modelling and drug screening applications. Here we established an efficient and robust 3D platform for cardiomyocyte (CMs) production from hiPSCs, solely through small-molecule-based temporal modulation of the Wnt signalling, which generates more than 90% cTNT^+^ cells. The impact of performing the differentiation process in 3D conditions as compared to a 2D culture system, was characterized by transcriptomic analysis by using data collected from sequential stages of 2D and 3D culture. We highlight that performing an initial period of hiPSC aggregation before cardiac differentiation primed hiPSCs towards an earlier mesendoderm lineage differentiation, via TGF-β/Nodal signaling stabilization. Importantly, it was also found that CMs in the 3D microenvironment mature earlier and show an improved communication system, which we suggested to be responsible for a higher structural and functional maturation of 3D cardiac aggregates.

## Introduction

Human embryonic stem cells (hESCs) and human induced pluripotent stem cells (hiPSCs), collectively referred as human pluripotent stem cells (hPSCs), offer an almost limitless source of cells for clinical translation applications. Particularly, cardiomyocytes (CMs) obtained from *in vitro* differentiation of hiPSCs have been considered an attractive tool for disease modelling and drug screening applications^1,2^.

The identification of key signalling pathways and the transcriptional network linked to embryonic heart development, guided the establishment of a number of *in vitro* models for cardiac differentiation from hPSCs. Through the sequential addition of growth factors and/or small molecules, the critical stages of cardiac specification have been recapitulated to some extent using 2D *in vitro* models^3–6^. However, human heart development is a complex process in which spatial gradients of molecules and biophysical stimuli, due to the three-dimensional (3D) configuration of the embryo, are crucial to determine the final heart tissue structure and function^7,8^. Therefore, these processes are not well recapitulated in the commonly used monolayer (2D) differentiation platforms.

Aiming at better mimicking the microenvironment of *in vivo* heart development, 3D platforms for *in vitro* cardiac differentiation and maturation have emerged in the past few years. However, and despite the existence of different reported protocols for hPSC differentiation into CMs as 3D aggregates^9–12^, the development of an efficient, controlled and reproducible process of 3D differentiation has been challenging.

3D culture of hiPSCs has been shown to favor transcriptional changes that improve differentiation into specific lineages^13–16^, but the mechanisms behind this effect have not yet been completely understood. Furthermore, the development of a platform that takes advantage of the reported knowledge regarding 3D culture of hiPSCs to establish a robust and straightforward cardiac differentiation protocol has not yet been reported. Additionally, the sole impact that 3D culture exerts throughout the process of hiPSC-CM differentiation, from the moment 3D aggregates are generated until the stage of CM maturation remains also poorly understood. As an example of the relevance of the culture format in this process, a recent study that performed 3D aggregation of cardiac progenitor cells obtained in 2D culture system, showed the benefits of 3D culture at earlier stages of cardiac differentiation regarding structural and metabolic maturation of the final CMs^17^.

In this work, we expanded the knowledge regarding the impact of 3D culture of hiPSCs in a forced aggregation platform and took advantage of that knowledge to develop a simple, efficient and robust 3D platform for hiPSC differentiation towards CMs, using the temporal modulation of the Wnt signalling pathway. RNA sequencing (RNA-seq) was used to generate global gene expression profiles for sequential stages of cardiac differentiation of both 3D aggregates and parallel monolayer 2D culture conditions. Expression profiling data analyses revealed that the initial period of hiPSC 3D aggregation before cardiac differentiation induces significant transcriptional changes that favour the cardiac differentiation process by priming hiPSCs to mesendoderm lineages. Also, the obtained data suggests that the CMs obtained in this 3D microenvironment mature earlier when compared with 2D cardiac monolayer.

## Results

### Forced aggregation of hiPSCs on microwells allows efficient generation of cardiomyocytes

**Figure 1:**
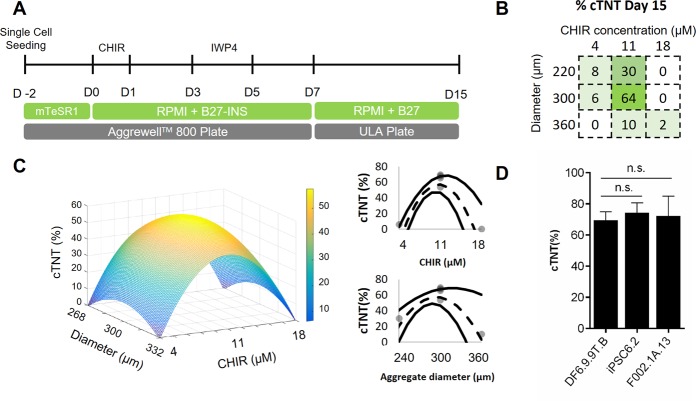
Forced aggregation of hiPSCs in microwells allows efficient generation of 3D cardiac tissue. (**A**) Schematic representation of cardiomyocyte differentiation from hiPSCs in a 3D culture system, using the temporal modulation of Wnt signalling. AggreWell™800 plates were used to obtain size-controlled aggregates. ULA – Ultra Low Attachment plates. (**B**) Percentage of cTNT^+^ cells after 15 days of differentiation for the experimental runs of the factorial design. CHIR concentration ranges from 4 µM to 18 µM (centre in 11 µM), and aggregate diameter between 220 µm and 360 µm (centred in 300 µm). Each of the tested combinations was performed once, excluding the centred point of the factorial design in which n = 4 independent experiments were performed. (**C**) 3D representation of the quadratic model relating initial aggregate diameter and small molecule CHIR concentration with the percentage of cTNT^+^ CMs after 15 days of differentiation. (**D**) Validation of the optimal conditions (aggregate size: 290 µM; CHIR concentration: 11 μM) with DF6.9.9 T.B cell line and two other hiPSC lines (F002.1 A.13 and iPSC6.2). Data are represented as mean ± SEM, n = 3 independent experiments for F002.1 A.13 and iPSC6.2 lines and n = 6 independent experiments for DF6.9.9 T.B cell line. n.s. – No statistically significant (p-value > 0.05). See also Fig. S1.

To develop a platform for 3D cardiac differentiation of hiPSCs, we used the temporal modulation of Wnt signalling pathway^18^ and a factorial design approach^19^ for the optimization process. In order to generate size-controlled aggregates, forced aggregation of single hiPSCs in the commercially available AggreWell^TM^ 800 plates was performed. Aggregate size was controlled using different cell seeding densities (Fig. S1A), and hiPSC aggregates were maintained during 48 hours in mTeSR^TM^1, before starting the differentiation process (D0) (Fig. 1A).

The cardiac differentiation process was optimized by testing the combined effect of small molecule CHIR concentration, ranging from 4 µM to 18 µM, and aggregate diameter at day 0, ranging from 220 µm to 360 µm, with the model centred in the culture condition corresponding to 11 µM CHIR and 300 µm, respectively. Cardiomyocyte differentiation efficiency was evaluated as the percentage of cells expressing the cardiomyocyte marker cardiac troponin T (cTNT) at day 15 of differentiation. The experimental results obtained for the different tested conditions are represented in Fig. 1B. The centred condition (11 µM CHIR, 300 µm) was the one that yielded the highest percentage of cTNT^+^ cells after 15 days of differentiation, resulting in an average of 64 ± 4% cTNT^+^ cells (Fig. 1B and Fig. S1B). From the quadratic model generated from the experimental results (Fig. 1C and Fig. S1C), only the quadratic term of CHIR concentration and aggregate diameter had statistical significance (Fig. S1D), which resulted in a maximum value for the considered output of the model (cTNT^+^ cells at day 15) for a specific aggregate diameter and CHIR concentration. The optimal condition that allowed reaching a percentage of cTNT^+^ cells between 50–66% was obtained when initiating the integrated process with an aggregate diameter of 289 ± 12 µm and a CHIR concentration of 10.8 ± 0.5 µM, which is in fact close to the centred point used in the factorial design.

The optimized cardiac differentiation platform was validated using the DF6-9-9T.B cell line, the one used for the factorial design optimization process, and two additional hiPSC lines, iPSC6.2 and F002.1 A.13, proving the reproducibility and robustness of the platform (Fig. 1D). Additionally, a time-course analysis of cardiac differentiation markers until day 20 was performed, confirming the normal progression of cardiac differentiation (Fig. S1E). In addition to the parameters that were optimized through the factorial design, it was also verified that by decreasing to 24 hours (D-1) the culture period before initiating the differentiation, the efficiency in terms of percentage of cardiomyocytes drastically dropped (Fig. S1F), reinforcing the importance of the 3D hiPSC aggregates expansion period.

### 3D culture of hiPSCs under pluripotency maintenance medium primes hiPSCs towards mesendoderm lineage

In order to reveal the main impact of 3D culture towards cardiac differentiation, a transcriptomic analysis was performed (Table S1), using 2D cardiac differentiation, based also on the temporal modulation of the Wnt signalling, as control^5,20^.

**Figure 2:**
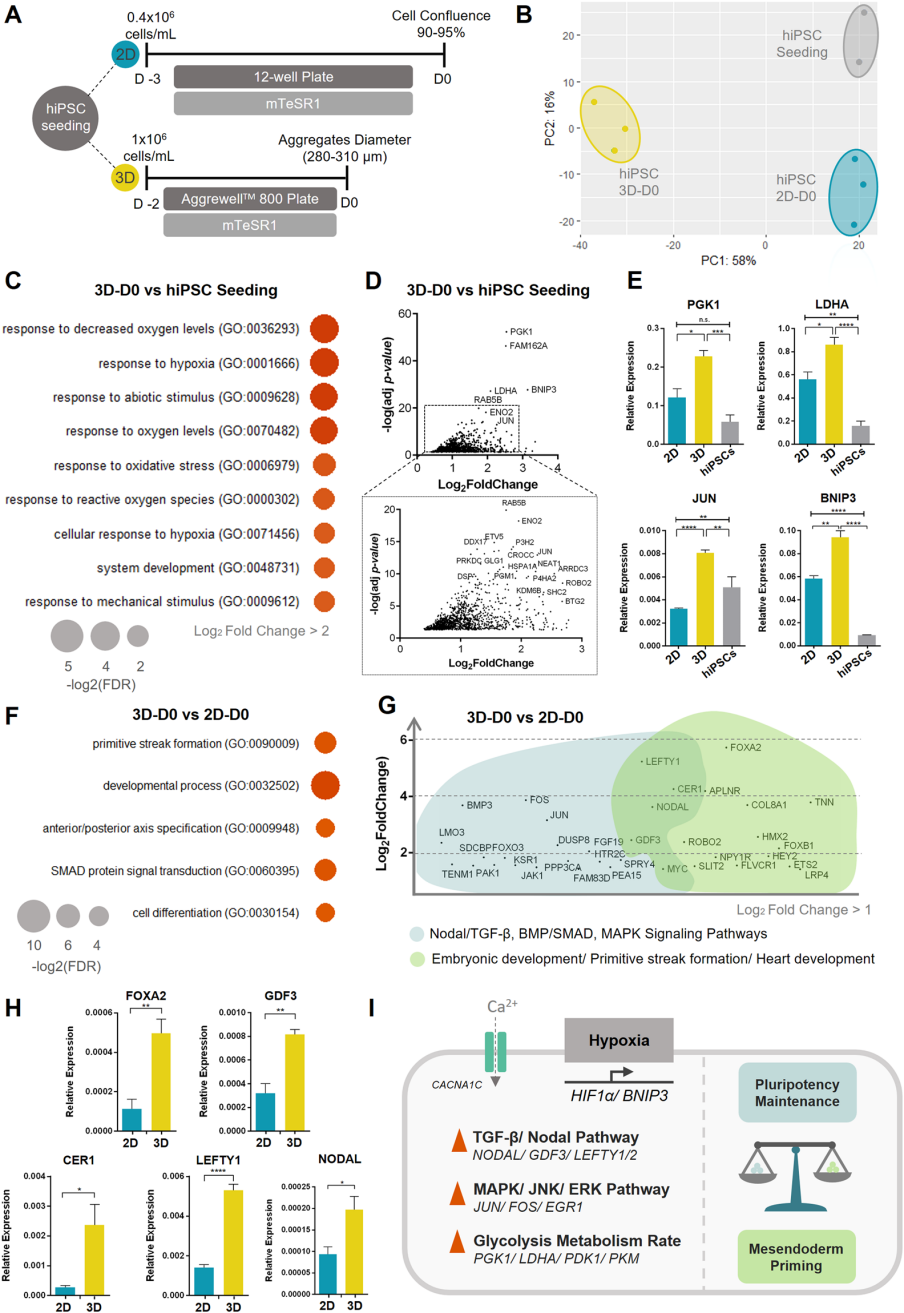
3D culture of hiPSCs under pluripotency maintenance medium primes hiPSCs towards mesendoderm lineage. (**A**) Schematic representation of hiPSC culture, before cardiac differentiation, in both 2D and 3D culture strategies. In 2D monolayer, hiPSCs were seeded at a density of 0.4 × 10^6^ cells/mL, reaching 90–95% confluence after 3 days of culture. In the 3D culture platform, hiPSCs were seeded at a density of 1 × 10^6^ cells/mL, reaching at day 0 of differentiation a diameter ranging from 280 to 310 µm. (**B**) PCA based on bulk RNA-seq data from three different conditions: hiPSCs used for seeding (hiPSC seeding, common for both culture strategies) (n = 2, independent experiments), 3D day 0 (3D-D0) and 2D day 0 (2D-D0) (n = 3, independent experiments). (**C**) Top gene ontology (GO) biological processes terms identified (FDR < 0.05) for the differentially upregulated genes (Log_2_ FC > 2 and adjusted *p-value* < 0.05) of 3D-D0 versus hiPSC seeding. (**D**) Volcano Plot highlighting the most significant upregulated genes for 3D-D0 versus hiPSC seeding populations (Log_2_ FoldChange > 1 and Adjusted *p-value* > 0.05). (**E**) Expression profile of *PGK1, LDHA, BNIP3* and *JUN* genes for the three different populations: 3D-D0, 2D-D0 and hiPSC seeding. Values are normalized to *GAPDH*. Data are represented as mean ± SEM, of at least n = 3 independent experiments. (**F**) Top GO biological processes terms identified (FDR < 0.05) for the differentially upregulated genes (Log_2_ FC > 2 and adjusted p-value < 0.05) of 3D-D0 versus 2D-D0 comparison. See Table S2 for full DE gene list. (**G**) Graphical representation highlighting a set of the upregulated genes in 3D-D0 versus 2D-D0 comparison, related with the enriched biological processes “Embryonic development/Primitive streak formation/Heart development” and “Nodal/TGF-β, BMP/SMAD and MAPK Signaling Pathways”, which stands out from the GO analysis (Log_2_ FoldChange > 1 and adjusted p-value > 0.05). (**H**) Expression profile of *FOXA2, GDF3, CER1, NODAL* and *LEFTY1* genes in 3D-D0 and 2D-D0 populations. Values are normalized to *GAPDH*. Data are represented as mean ± SEM, of at least n = 3 independent experiments. (**I**) Schematic representation of the proposed signalling network involved in hiPSCs culture as 3D aggregates in a forced aggregation platform. Oxygen gradients inside the spheroids trigger a hypoxic stimuli which is responsible for cell adaptation through (1) stabilization of TGF-β/Nodal pathway, (2) upregulation of MAPK/JNK/ERK pathway and (3) increase in glycolysis metabolism, culminating in a balance between pluripotency maintenance and hiPSCs priming towards differentiation, particularly into mesendoderm lineage (Fig. 2I). See also Fig. S2.

The first stage of hiPSC culture before cardiac induction involves a short period of hiPSCs expansion for both 2D and 3D culture conditions (Fig. 2A), which from now on will be designated as the pre-differentiation period. As confirmed by principal component analysis (PCA) (Fig. 2B), gene expression profile of D0 hiPSC population for both 2D (“2D-D0”) and 3D (“3D-D0”) differentiation culture formats, showed considerable differences when compared with the “hiPSC seeding” population (initial hiPSCs seeded in 2D and 3D platforms), mainly discriminated by PC2 (16% of total variance), which is probably related with the degree of cell confluence and/or cell communication. Differences at gene expression level between “3D-D0” and “2D-D0” are also evident, and were mainly discriminated by PC1 (58% of total variance), which explains the majority of the observed differences in the analysed dataset and seems to be linked with the culture format (Fig. 2B and Fig. S2A).

To understand those differences, a comparative analysis between “3D-D0” and “hiPSC seeding” expression profiling was performed (Table S2 “DE gene list”). Gene ontology (GO) analysis using the upregulated genes shows that the most enriched biological process in the 3D culture format was related with response to hypoxia (Fig. 2C and Table S2 “GO - 3D D0 vs hiPSC seeding - up”). Since 3D hiPSC aggregates had already ≈290 µm of diameter at D0, it is not surprising the activation of genes involved in hypoxic response as a result of oxygen diffusion limitations throughout the entire aggregate^21^. To demonstrate the existence of an hypoxic environment inside the 3D aggregates, the expression level of the HIF1α protein, which is the master transcriptional regulator of hypoxic response^22^, was quantified in both “2D-D0” and “3D-D0” cell populations. The results revealed a significant increase in HIF1α protein expression levels in 3D aggregates as compared to the 2D monolayer (Fig. S2B). Since gradients of oxygen and nutrients inside the 3D aggregates could potentially compromise cell viability, we quantified the percentage of viable cells in 3D-D0 aggregates and compared with “2D-D0” population, confirming that the viability of the cells was not affected by 3D culture (Fig. S2C).

To highlight the genes that were upregulated in the “3D-D0” cell population vs “hiPSC seeding” population, a volcano plot was generated (Fig. 2D). Among the upregulated genes, *PGK1*, *LDHA*, *BNIP3* and *JUN* stand out, within the group of genes that show a higher and more significant upregulation. The higher expression level of those genes in 3D-D0 aggregates, when compared not only to “hiPSC seeding” cells but also to 2D-D0 monolayer cells, was confirmed by qRT-PCR, (Fig. 2E). In agreement with the hypothesis of an hypoxic response inside the 3D aggregates, the aforementioned genes are known direct or indirect targets of HIF1α activation network. Specifically, *PGK1* and *LDHA* are genes involved in glycolysis, which suggests that in the 3D environment the rate of glycolysis is higher compared with both “2D-D0” and “hiPSC seeding” conditions. GO analysis of the downregulated genes in “3D-D0” and “2D-D0” environments compared to “hiPSC seeding” population, revealed that some of the downregulated genes in 3D-D0 population are related with the oxidative phosphorylation and mitochondrial respiratory chain (Fig. S2D,E), reinforcing the proposal of a higher rate of glycolysis in 3D-D0 aggregates. Finally, glucose consumption and lactate production rates were analysed in both “2D-D0” and “3D-D0” conditions. The results revealed an equivalent specific glucose consumption rate in 2D-D0 and 3D-D0 conditions, however a statistically significant higher specific lactate production rate in hiPSCs present in the 3D-D0 aggregates was observed when compared with the cells in 2D monolayer. This results on a higher yield of lactate production/glucose consumption, which is indicative of a higher level of glycolysis in 3D-D0 aggregates (Fig. S2F). In fact, hypoxia is a known trigger of metabolic changes in hPSCs and it is also described to be involved in pluripotency maintenance by promoting glycolysis and preventing mitochondrial respiration^23,24^.

The activation of MAPK/JNK/ERK signalling is also evident in “3D-D0” aggregates when compared with “hiPSC seeding” and “2D-D0”, with the upregulation of *JUN, FOSB, FOS and EGR1* genes. The possible activation of this pathway, which is involved in the transcription of a wide range of cell proliferation and apoptotic genes^25^, can be also a consequence of reduced oxygen levels, specifically due to increased concentration of ROS inside the cells^26^. Additionally, JNK/ERK signalling pathway can be indirectly activated as a result of the increased cytosolic concentration of Ca^2+^ triggered by ROS^27^, since Ca^2+^ has been described to be involved in MAPK/JNK/ERK signalling activation^28^. In fact, the calcium voltage channel Cav1.2 encoded by *CACNA1C*, is upregulated in 3D-D0 aggregates, as well as *CXCR4*, which is described to be involved in the regulation of Ca^2+^ mobilization and activation of MAPK signalling^29^. MAPK/JNK pathway had been reported to be involved in the maintenance of stemness of hPSCs but activation of this pathway has also been reported to be linked with the initiation of hPSC differentiation^30^.

Additionally, a differential gene expression analysis between “3D-D0” and “2D-D0” was performed (Table S2 “DE gene list”). From the GO analysis focused on the most significantly upregulated genes, the main differences were related with regulation of Nodal signalling, SMAD protein signal transduction and the induction of primitive streak (PS) (Fig. 2F and Table S2 “GO - 3D-D0 vs 2D-D0 - up”). Within the upregulated genes, ligands of Nodal signalling, including *NODAL* and *GDF3*, as well as their direct targets, *LEFTY1*, *LEFTY2* and *CER1*, that act in a negative feedback loop^31^, stand out (Fig. 2G). The upregulation of some of these genes, namely *GDF3*, *CER1*, *LEFTY1* and *NODAL*, was confirmed by qRT-PCR, corroborating the RNA-seq data (Fig. 2H). Interestingly, Nodal signalling is not upregulated in “3D-D0” vs “hiPSC seeding” and instead it is down-regulated in “2D-D0” vs “hiPSC seeding”, meaning that 3D culture of hiPSCs potentially induced the stabilization of Nodal signaling.

Although Activin/Nodal signalling is described to be involved in pluripotency maintenance of hPSCs, it is also described to be responsible for driving early cell fate decisions along the mesendodermal lineages^31^. The upregulation of *FOXA2* in “3D-D0” aggregates compared with “2D-D0” (Fig. 2H), which is expressed in early/anterior PS^32^, combined with TGF-β/Nodal signalling activity, strengthens the hypothesis of hiPSC priming to mesendoderm lineages in 3D conditions.

In summary, our results suggest that hiPSCs culture as 3D aggregates in a forced aggregation platform results in oxygen gradients inside the spheroids, which are responsible for cell adaptation through (1) stabilization of TGF-β/Nodal pathway, (2) upregulation of MAPK/JNK/ERK pathway and (3) increase in glycolysis metabolism, culminating in a balance between pluripotency maintenance and hiPSC priming towards differentiation, particularly into mesendoderm lineage (Fig. 2I).

### 3D cardiac differentiation allows a faster structural and functional maturation of hiPSC-CMs when compared with the 2D platform

**Figure 3:**
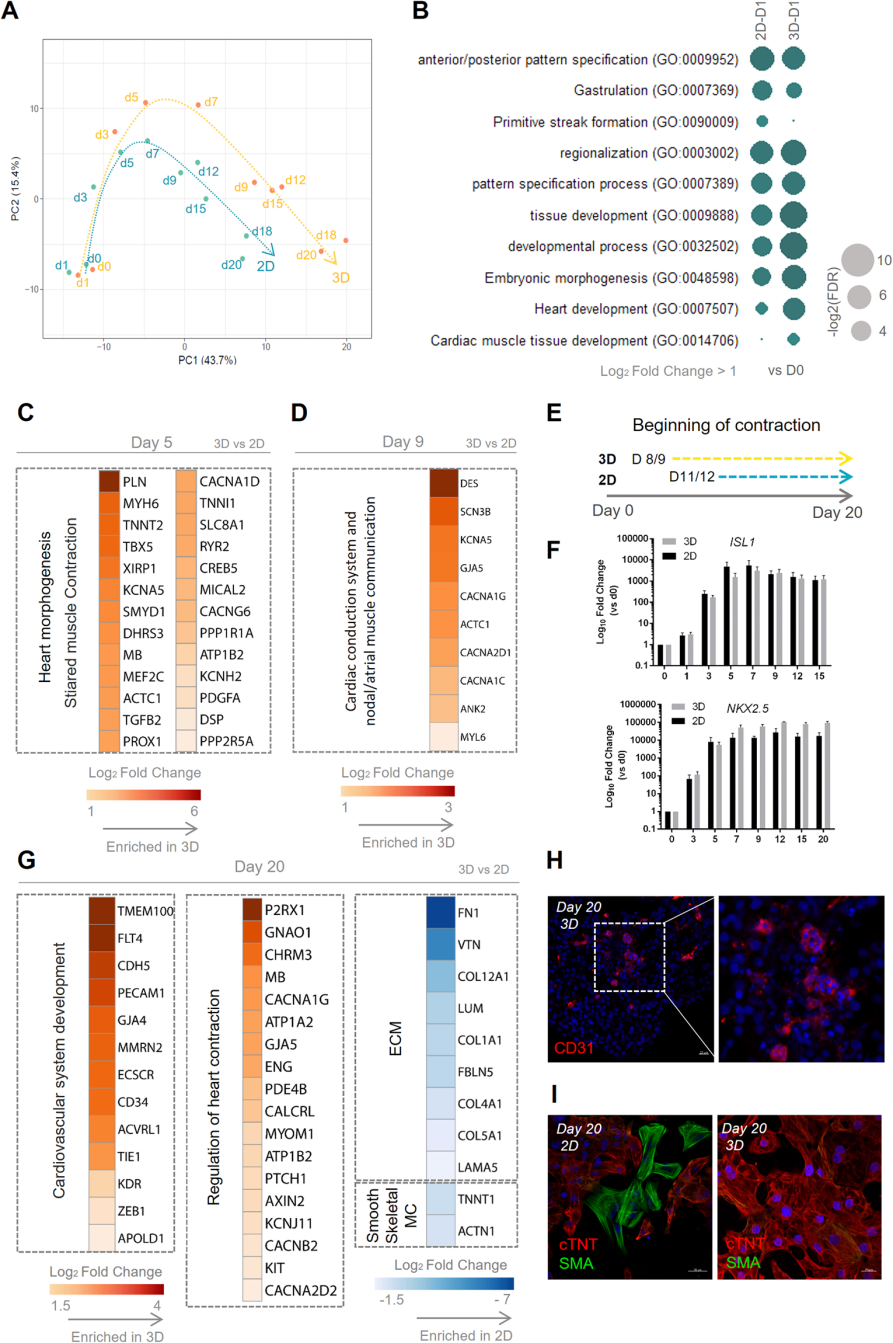
3D cardiac differentiation allows a faster structural and functional maturation of hiPSC-CMs when compared with the 2D platform. (**A**) PCA of RNA-seq data in counts per million (CPM) using a subset of 254 genes linked to cardiac differentiation progression, CM functional and structural maturation, CM metabolism, Wnt, TGF-β and FGF signalling pathways and other cardiac cells. See also Table S3 for more details. Orange and blue arrows describe the pathway followed by 3D and 2D cardiac differentiation, respectively, until day 20 of differentiation. (**B**) Top GO terms for biological processes identified (FDR < 0.05) for differentially upregulated genes (Log_2_ FC > 1 and adjusted *p-value* < 0.05) for 3D-D1 versus hiPSC seeding, and 2D-D1 versus hiPSC seeding. (**C,D**) Heat map highlighting the differentially expressed genes (Log_2_ FC > 1 and adjusted p-value < 0.05) related with the most significant GO terms for (**C**) 3D-D5 vs 2D-D5 and (**D**) 3D-D9 vs 2D-D9. See Table S3 for full DE gene list. (**E**) Schematic representation highlighting the moment of begging of contraction in both 3D aggregates and 2D monolayer. (**F**) Expression profile of cardiac progenitor genes *ISL1* and *NKX2-5* throughout the entire process of cardiac differentiation. Values are normalized to *GAPDH* and relative to day 0. Data are represented as mean ± SEM, n = 3 independent experiments. (**G**) Heat map highlighting the differentially expressed genes (Log_2_ FC < -1 and Log_2_ FC > 1) and adjusted p-value < 0.05) related with the most significant GO terms for 3D-D20 vs 2D-D20 differential expression analysis. See Table S3 for full DE gene list. (**H**) Representative section of 3D aggregates on day 20 of differentiation, highlighting the presence of CD31^+^ cells. Scale bars, 20 µm. (**I**) Replated CMs from dissociated 3D aggregates and 2D cardiac tissue at day 20 of differentiation, highlighting the prevalence of SMA^+^ cells in the 2D culture when compared to 3D conditions. Scale bars, 50 µm and 20 µm in 2D and 3D conditions, respectively. See also Fig. S3.

To understand the impact of hiPSC 3D culture during the process of cardiac differentiation, we analysed the differentially expressed genes in 3D aggregates throughout the differentiation process, when compared with the 2D monolayer differentiation. Using a set of known genes involved in cardiogenesis and cardiomyocyte maturation, a PCA analysis (Table S3 “PCA 3D and 2D (D0 -D20)”) was performed, which highlights differences regarding the progression of cardiac differentiation in both culture systems (Fig. 3A).

During the first 7 days of cardiac differentiation, 3D aggregates remain inside the microwell platform, which seems to be responsible for a prolonged oxidative stress and activation of hypoxic response. The transcriptional activation of metallothionein (MT) protein-coding genes during the first days of 3D cardiac differentiation, including *MT1G*, *MT1H*, *MT1E*, *MT2A*, *MT1F* and *MT1X*, which prevent cells from apoptosis^33^, reinforce the hypothesis of an oxidative stress response.

At day 1 of differentiation, GO analysis focused on the most significantly upregulated genes in 3D-D1 aggregates and 2D-D1 monolayer when compared to “3D-D0” and “2D-D0” initial populations, respectively (Fig. 3B), revealed that the most upregulated genes in “3D-D1” were statistically more related with “tissue development process” and “cardiac tissue specification” biological processes, and in “2D-D1” more related with “gastrulation”, “primitive streak formation” and “anterior/posterior pattern specification” biological processes. This corroborates the idea that 3D aggregates at day 0 of differentiation are more primed for mesendoderm differentiation and, consequently, cardiac differentiation progresses faster. Focusing our analysis only on transcription factors (TFs), RNA-seq data indicates that TFs known to be related with cardiac differentiation, such as *MESP1*, *GATA4*, *TBX3*, *MSX2*, were already upregulated at D1 of differentiation in 3D aggregates (Fig. S3A). Additionally, a higher expression of the gene *T*, which is one of the most important genes involved in mesendoderm specification, in 3D-D1 aggregates when compared with 2D-D1 monolayer, was further confirmed by qRT-PCR analysis (Fig. S3B). Also, the Wnt/β-catenin target genes, *AXIN2* and *DKK1*, were statistically significantly upregulated in the 3D aggregates compared with 2D monolayer at day 1 of differentiation, suggesting a higher degree of canonical Wnt signalling activation (Table S3 “DE gene list”).

Moving along the cardiac differentiation process, at day 5 of differentiation a set of genes related with cardiac development and sarcomere structure and function were upregulated in the 3D aggregates (Table S3 “DE gene list”), including *MEF2C*, *MYH6*, *ACTC1*, *ATP1A2, CACNA1D, RYR2, TNNT2 and TNNI1* (Fig. 3C). At day 9 of differentiation, the differential expression analysis between 3D and 2D cardiac differentiation conditions (Table S3 “DE gene list”), revealed an enrichment in 3D aggregates of GO terms related with cardiac conduction system and nodal/atrial muscle communication. Specifically, the protein-coding genes *CACNA1C*, *GJA5*, *CACNA1G, SCN3B, KCNA5* and *ANK2* were upregulated (Fig. 3D). In fact, most of these genes are described to be present in atrial CMs or in the nodal/conduction system myocytes. *GJA5* gene, that encodes for the Cx40 gap junction, and *KCNA5*, that encodes for the ion channel Kv1.5, are described as cell markers for atrial CMs^34,35^, being almost absent in the ventricular working myocardium. Additionally, *GJA5* is largely expressed in the conduction system myocytes, which includes the HIS bundles, the left and right bundle branches (LBB and RBB) and purkinje fibers, enabling a fast conduction of the impulse between the atrioventricular node (ANV) and ventricular working myocytes^36^. The calcium channel Cav3.1, encoded by *CACNA1G*, is preferentially expressed in nodal/pacemaker myocytes although it is also abundantly expressed in atrial CMs and purkinje fibers^37,38^. Interestingly, it is at day 8/9 of differentiation that 3D aggregates start to contract, in contrast with 2D monolayer culture, in which the beginning of contraction only starts at day 11/12 of differentiation (Fig. 3E). Despite this apparent earlier expression of structural and functional CM genes in 3D aggregates, RNA-seq data did not show a delay in the expression of cardiac progenitor markers, namely *ISL1* and *NKX2-5*, in 2D monolayer vs 3D aggregates, a result further confirmed by qRT-PCR analysis (Fig. 3F).

At day 15 of differentiation, from a GO analysis of genes that were only upregulated in 3D aggregates or 2D monolayer, it was possible to observe that, in the case of 3D aggregates, regulation of heart contraction and cardiovascular system development appear as upregulated biological process, whereas in the case of 2D monolayer those terms were not represented and instead terms related with extracellular matrix organization and structure were highlighted (Fig. S3C).

**Figure 4:**
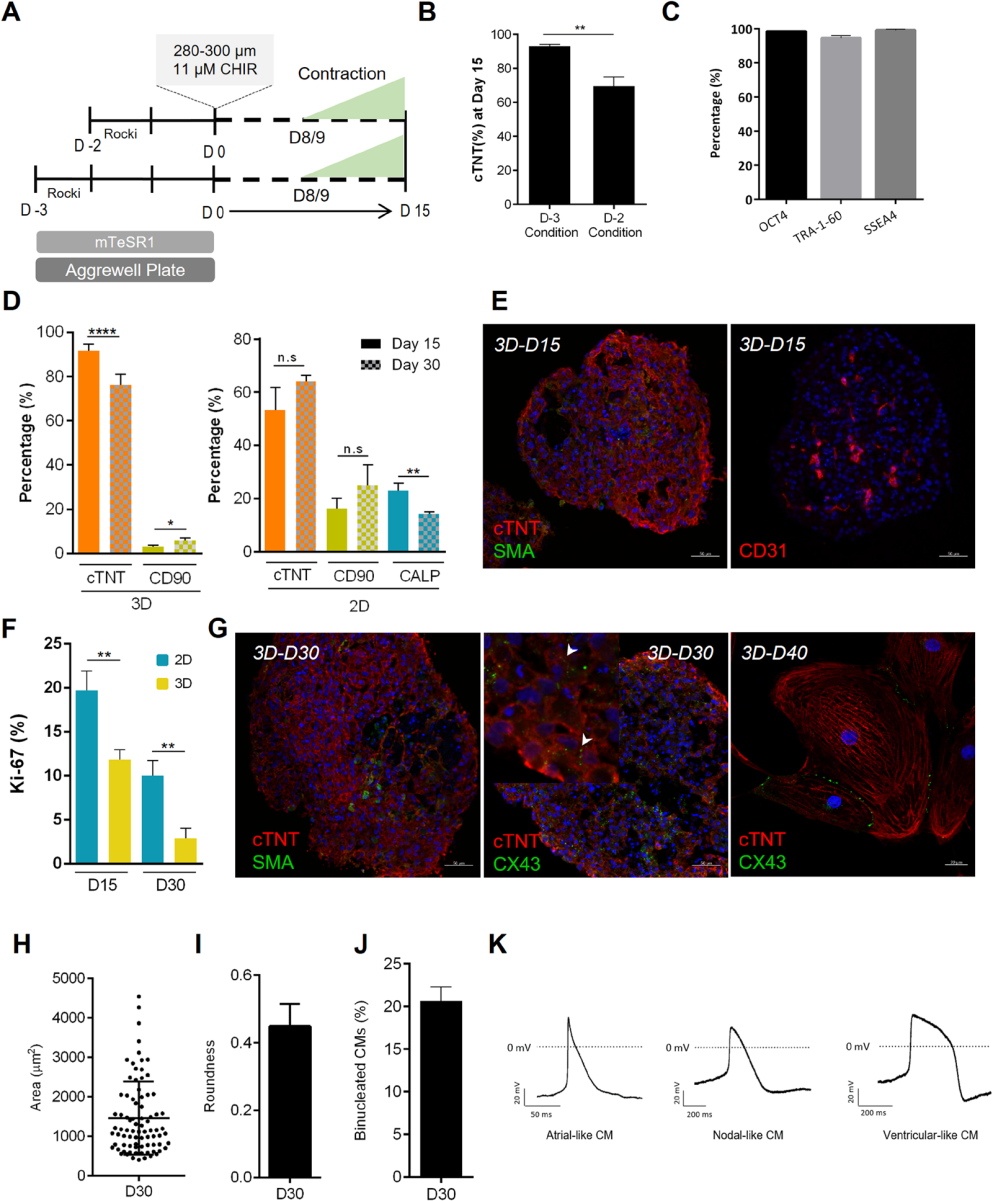
Performing the cardiac differentiation in 3D conditions impacts the cellular composition of cardiac aggregates and the maturity of cardiomyocytes. (**A**) Schematic representation highlighting the optimized 3D cardiac differentiation platform, in which hiPSC aggregates remain for 3 days in pluripotency maintenance medium before starting the differentiation process. The aggregate diameter at D0 was the same obtained with the previous version of the 3D platform (pre-differentiation period of 2 days) since the initial cell seeding density was decreased. (**B**) Cardiomyocyte differentiation efficiency in terms of cTNT^+^ cells after 15 days of differentiation in both conditions, pre-differentiation period of 2 days (D-2) or 3 days (D-3). Data are represented as mean ± SEM, n = 6 independent experiments. (**C**) Flow cytometry analysis of hiPSC 3D aggregates at D0 of differentiation, after 3 days in pluripotency maintenance media, for pluripotent transcription factor OCT4, and surface markers TRA-1-60 and SSEA4. Data are represented as mean ± SEM, n = 3 independent experiments. (**D**) Quantification of different cell types present in 2D monolayer and 3D aggregates at D15 and D30 of differentiation (cTNT^+^ - CMs; CD90^+^ - Fibroblast-like cells; CALP^+^ - Smooth Muscle cells). Data are represented as mean ± SEM, at least n = 3 independent experiments. (**E**) Sections of 3D aggregates at day 15 of differentiation. Scale bars, 50 µm. (**F**) Percentage of CMs-Ki67^+^ cells in 3D aggregates and 2D monolayer at D15 and D30 of differentiation. Data are represented as mean ± SEM, n = 3 independent experiments. (**G**) Sections of 3D aggregates at D30 of differentiation and D40 replated CMs. Scale bars, 50 µm, for sections, and 20 µm in replated CMs. (**H–J**) Characterization of D30 replated CMs in terms of CMs area (**H**), roundness (**I**) (data are represented as mean, n = 3 (82 cells) independent experiments), and binucleation (**J**) (data are represented as mean, n = 3 (317 cells) independent experiments). (**K**) Representative traces of action potential (AP) recordings in day 30–35 of replated 3D aggregates of CMs, using whole cell patch clamp. n = 3 independent experiments. See also Fig. S4.

A differential expression analysis comparing both cardiac tissues at day 20 of differentiation (Table S3 “DE gene list”, “GO - 3D-D20 vs 2D-D20 - up”) revealed an upregulation of genes related with endothelial differentiation/endothelium formation, such as *KDR*, *CDH5*, *PECAM1*, *CD34* and *GJA4* (Fig. 3G) in the 3D aggregates. The presence of CD31^+^ endothelial cells in 3D aggregates was further validated by immunostaining of aggregate sections (Fig. 3H). In addition, genes involved in heart contraction, action potential and signal conduction, such as *KCNJ11*, *GJA5*, *CACNA2D2*, *CACNA1G*, *ATP1A2, MYOM1* and *MB*, were also upregulated in the 3D aggregates, suggesting a higher degree of functional maturation. In the 2D monolayer cardiac tissue, different protein-coding genes of ECM were upregulated, including different collagens (*COL1A1, COL4A1, COL5A1, COL9A1*) lumican (*LUM*), laminin (*LAMA5*), fibronectin (*FN1*) and vitronectin (*VTN*). The upregulation of ECM protein-coding genes throughout the process of cardiac differentiation was already reported for 2D monolayer cardiac differentiation^39^, as well as the upregulation in 2D monolayer compared with 3D aggregates^17^. The increased expression of these genes can be related with a higher content of fibroblast-like cells in the 2D monolayer since they are the main producers of ECM^40^. In addition, in 2D cardiac tissue, expression profiling results suggested the upregulation of protein-coding genes for skeletal muscle myocytes, namely *TNNT1*, and the upregulation of *ACTN1* and *TAGLN2*, which suggests higher content of smooth muscle cells compared with the 3D cardiac aggregates. In fact, immunostaining of replated 3D aggregates and 2D monolayer suggests a higher prevalence of smooth muscle cells in the 2D culture system (Fig. 3I), which was further confirmed by CALP^+^ cell quantification (Fig. 4D).

### Performing the cardiac differentiation in 3D conditions impacts the cellular composition of cardiac aggregates and the maturity of cardiomyocytes

Taking into consideration the results obtained from the RNA-seq data regarding the transcriptional effect of 3D culture of hiPSCs prior to cardiac differentiation, we tested the effect of increasing the pre-differentiation period from 2 to 3 days, while maintaining the optimized D0 aggregate size (Fig. 4A). This increment resulted in a significant increase of CM differentiation efficiency from ±70% to >90% cTNT^+^ (92 ± 1%) cells after 15 days of differentiation (Fig. 4B), without compromising the expression of pluripotency markers OCT4, SSEA4 and TRA-1-60, at D0 (Fig. 4C). Also, increasing the pre-differentiation period improved the reproducibility among biological runs, which is still a common bottleneck in different reported studies of 2D cardiac differentiation, mainly the ones based on Wnt signalling modulation^41^.

The 3D cardiac spheroids obtained with this improved 3D cardiac differentiation platform were then further characterized in terms of cell composition and compared to 2D monolayer. In 3D aggregates, apart from CMs, a small population of non-myocyte cells were detected at D15, comprising mainly CD90^+^ stromal cells (3.3 ± 0.1% CD90^+^ cells) (Fig. 4D). Additionally, immunostainning of different sections of cardiac aggregates shows the presence of different areas staining positively for the endothelial marker CD31 (Fig. 4E), demonstrating the existence of endothelial cells inside the aggregates. At day 30, aggregates show a decreased percentage of cTNT^+^ cells, which can be attributed to the increased number of proliferative cells, particularly CD90^+^ fibroblast-like cells, which percentage increased to ±6% at D30 (Fig. 4D). However, it can also be attributed to the observed decrease in total cell number (±20% of cell loss) from D15 to D30 of differentiation, which potentially results from aggregates agglomeration. To avoid this phenomenon, two different modifications to the initial method were evaluated including (i) single-cell dissociation of the 3D aggregates at D10 of differentiation followed by re-aggregation in 96 well plates or (ii) direct transfer of the 3D aggregates to 96-well plates (Fig. S4A). Both approaches allowed the maintenance of viable 3D aggregates until D30 of cardiac differentiation (Fig. S4B,C). Comparing with 3D culture format, the efficiency of 2D cardiac differentiation was lower (55 ± 5% cTNT^+^ cells) at D15 and a higher variability between biological runs was observed (Fig. 4D). In agreement with RNA-seq data, a higher prevalence of fibroblast-like cells (16 ± 2% CD90^+^ cells) and smooth muscle cells (23 ± 2% CALP^+^ cells) was also confirmed in the 2D cardiac differentiation platform (Fig. 4D). Additionally, 2D cardiac monolayer differentiation lead to the detachment of contracting areas from the surface of culture matrix approximately after 20 days of culture, resulting in a considerable cell loss (±50%) and consequently reduced cell yield and higher variability. Additionally, using cTNT to specifically label cardiomyocytes and Ki67 as a marker of cell proliferation, we demonstrate the decrease in CMs-Ki67^+^ cells in both culture formats from D15 to D30. However, 3D aggregates show a significantly lower percentage of CMs staining positively for Ki67 at both analysed time points, decreasing from 11.9 ± 0.6% at D15 to 2.9 ± 0.7% at D30, whereas in 2D it decreases from 19.7 ± 1.2% at D15 to 10.0 ± 1% at D30 (Fig. 4F). This progressive exit from the cell cycle during maturation in 3D suggests a more mature phenotype compared to the one obtained in the 2D monolayer. Importantly, the expression of TNN13, which represents the more mature isoform of cardiac troponin TNNI^42^, is higher in 3D aggregates compared to 2D monolayer at D15 of differentiation (Figure S4D), reinforcing the idea of a higher degree of CM maturation in 3D cardiac aggregates at this time point.

After 4 weeks in culture, CMs from 3D cardiac aggregates show positive staining for connexin 43 (Cx43) between neighbour CMs, which is an important gap junction present in the working myocardium (Fig. 4G). Additionally, CMs replated from D30 aggregates show an average area of ±1,456 µm^2^ and a roundness of ±0.45, with 20.6 ± 1.6% of binucleated CMs (Fig. 4H–J), which is in agreement with previous reports of CM in 3D culture^43,44^. Finally, using the patch clamp technique, it was also possible to validate the electrophysiological activity of CMs dissociated from aggregates at day 30 of differentiation (Fig. 4K).

## Discussion

When compared to 2D culture systems, 3D differentiation of hPSCs has proved to better mimic the process of embryogenesis *in vivo*, by recreating important spatial gradients of different signals that are essential for normal embryonic development, particularly for cardiogenesis^14^. Previous studies in the literature have mainly explored the implication of the 3D environment in cardiac tissue maturation, starting with a population of cardiac progenitor cells^17^ or CMs previously differentiated in 2D monolayer culture systems^44–46^. In a complementary approach to these studies, here we revealed important implications involved in transitioning the Wnt signalling-based 2D culture system to our novel integrated 3D culture platform, starting with forced and controlled aggregation of hiPSCs followed by their expansion, cardiac differentiation and maturation, in a 3D environment.

Oxygen gradients have been described as a fundamental physiological cue during organogenesis in the developing embryo^47^. In fact, due to oxygen diffusional limitations and also due to a non-established circulatory system, the development of the embryo in the early stages of embryogenesis occurs in a relatively oxygen-poor environment^48^. In our 3D culture platform, aggregates at day 0 of differentiation have already around 300 µm of diameter, which can result in oxygen gradients since oxygen diffusion distance is estimated to be approximately 150 µm^21^.

In this work we demonstrated that 3D culture of hiPSC before cardiac differentiation induced important regulatory changes that influenced the efficiency and robustness of cardiac differentiation. We attributed the 3D culture format itself and the consequent hypoxic response generated inside the aggregates as the major triggers for the transcriptional changes that we observed, namely (1) the stabilization of TGF-β/Nodal pathway, (2) the upregulation of MAPK/JNK/ERK pathway and (3) the increase in glycolysis energy metabolism, which culminates in a balance between pluripotency maintenance and hiPSCs priming towards mesendoderm lineage differentiation. In fact, in the literature there are already evidences that suggest the priming of hPSCs towards mesoderm when cultured as 3D aggregates^13,15^. Also, *HIF1α* has been described to indirectly induce *NODAL* transcription via Notch signal stabilization^49,50^, which can explain TGF-β/Nodal pathway stabilization in our 3D hiPSC aggregates. The induction of mesoderm, during heart development, begins with high concentrations of *NODAL* in the proximal epiblast on mouse embryos^51^. Additionally, *in vitro* studies revealed that canonical Wnt and TGF-β/Nodal signalling work together in the regulation of PS formation and suppression of *NODAL* prevents β-catenin mediated PS induction by CHIR addition^52^, reinforcing the importance of the Nodal signalling during the early stages of cardiac differentiation. Here we demonstrated that the initial stage of hiPSC culture as 3D hiPSC aggregates, before cardiac induction, influences the progression of differentiation, culminating in a faster and more efficient commitment of hPSCs towards cardiac mesoderm and consequently towards cardiomyocytes. We attribute this faster progression of cardiac differentiation in 3D aggregates, compared with 2D monolayer, not only to the differences in the initial cell population but also to the continuous hypoxic stimuli that may still be present throughout the differentiation process and the 3D structure itself, with increased cell-to-cell interaction.

Kinney and collegues^53^ referred in their work that forced aggregation allows a more homogeneous control of intercellular adhesion dynamics which may impact the differentiation capacity of ESCs. Particularly, they explored the relationship between E-cadherin, linked to cell-cell interaction, and β-catenin, involved in canonical Wnt signalling, which may be responsible for an enhanced cardiogenic differentiation capacity in 3D-EB-like aggregates. This fact can explain our RNA-seq data suggesting a higher degree of Wnt signaling activation in 3D aggregates at early stages of cardiac differentiation and also the upregulation of important targets of the Wnt/β-catenin pathway, such as *MESP1* and *MEF2C*. Additionally, previous studies suggest that hypoxia enhances the expression of mesodermal genes, acting as a mesoderm-inductive signal^54^, and also promote the activation of Wnt/β-catenin signalling pathway^55^. Additionally, hypoxia, through *HIF1α* stabilization, has been suggested to have, as direct targets, important cardiac transcriptional factors, such as *MEF2C*^56^, which was upregulated in our 3D aggregates, and be also involved in the process of myofibrillogenesis^56^. In fact, different genes involved in cardiomyocyte structural and contraction apparatus, and also cardiac communication, including different gap junctions and ion channels genes, were upregulated at early stages of differentiation in 3D aggregates. This can potentially explain the earlier beginning of contraction in the 3D culture system compared with 2D monolayer.

Zhang and colleagues^12^, which also studied in parallel 2D and 3D cardiac differentiation systems, concluded that no major differences in the kinetics of cardiac differentiation became apparent between the 2D and 3D formats, suggesting a synchronized differentiation of the cells in both conditions. In that particular study, aggregates were induced at the same day that differentiation starts, contrary to what happens in our platform, where a pre-differentiation period is integrated with the differentiation platform and, as discussed before, has significant impact on the progression of differentiation. Also, Kerscher and colleagues^57^, which compared the differentiation into CMs from hPSCs encapsulated in a 3D hydrogel structure with a 2D monolayer culture system, reported that CM yield and gene expression level of cardiac markers were analogous to the ones observed in 2D monolayer for the same analysed time points. This reinforces the relevance and novelty of the integrated 3D cardiac differentiation platform that we developed.

Interestingly, some of the upregulated genes during the progression of differentiation and in the final 3D cardiac spheroids, compared with the 2D culture system, are involved in cardiac cell communication, and some of them are described to be preferentially expressed in atrial, nodal and conduction system CMs. This suggests that different and/or more functional CMs of different subtypes might be present in the 3D aggregates compared with the 2D monolayer. Since different subtypes of CMs are described to be originated from different subtypes of cardiac progenitor populations^35^, we suggest that the 3D environment is more prone to the development of a heterogeneous cardiac differentiation environment as a result of spatial gradients of molecules and oxygen inside the aggregates. In 2D monolayer culture systems cells are more homogenously exposed to the different stimuli, which can result in a more homogeneous differentiation towards a specific subtype of CMs. Taking into consideration the RNA-seq data, we cannot claim the existence of a different proportion or an enrichment in a specific CM subtype in each culture condition. However, a recently identified surface marker for ventricular-like cardiomyocyte progenitor cells, *LIFR*^58^, was upregulated (FC ± 2.7) in the 2D culture at day 7 of differentiation compared to 3D aggregates. In fact, the 2D differentiation protocol based solely on the temporal modulation of Wnt signalling, has already been described to bias the cardiomyocyte differentiation towards ventricular-like cardiomyocytes^59^. A single-cell RNA-seq approach could enable the identification and characterization of the different cardiac progenitor populations that are potentially developed at the early stages of differentiation and of the different subtypes of cardiomyocytes present in the final 3D aggregates.

In conclusion, we developed a simple, highly efficient and robust 3D cardiac differentiation platform, using only the temporal modulation of Wnt signalling. This 3D integrated hiPSC expansion and differentiation platform contributes to a faster cardiac commitment of hiPSCs and to an earlier CM structural and functional maturation when compared with CMs obtained from monolayer culture. 2D differentiation of hiPSCs into cardiomyocytes using the Wnt signalling modulation, despite being a simple protocol, is a process that has a very high intrinsic variability^41^ ending up to be a poorly reliable process when the aim is to obtain CMs in a consistent manner for further applications. CMs produced with our 3D platform can be easily used for the development of *in vitro* engineered cardiac tissue (EHT) models, which in the majority of the reported cases uses hPSC-CMs previously differentiated in 2D culture platforms. With our 3D differentiation platform, hiPSC-CMs can be obtained in a faster, more efficient and reproducible way, and additionally CMs show already a higher degree of maturation compared to age-matched CMs obtained in 2D culture. The 3D platform allows the achievement of 20–25 million CMs at D10-D15 of cardiac differentiation per AggreWell^TM^800 plate, and overall this number can be easily increased using a scale out process.

## Experimental Procedures

### Cell maintenance

Human iPSCs were maintained in mTeSR^TM^1 medium (StemCell Technologies) on Matrigel-coated (Corning), tissue culture plates. Medium was changed daily. Cells were routinely passaged every three to four days using 0.5 mM EDTA solution (Thermo Fisher Scientific).

### Cardiomyocyte differentiation in 2D and 3D culture conditions

For 2D monolayer culture, hiPSCs were seeded onto Matrigel-coated 12-well tissue culture plates and cultured in mTeSR^TM^1. Culture medium was changed daily until a confluence of around 90–95% was attained. For 3D aggregates formation, hiPSCs were seeded on microwell plates (AggreWell™800, StemCell Technologies) according to the manufacturer’s instructions. For hiPSCs differentiation into cardiomyocytes, in both 2D and 3D culture conditions, an adapted GiWi protocol was used^5^. RPMI 1640 medium (Thermo Fisher Scientific) was used as basal medium. From day 0 to day 6, cells were cultured in RPMI medium supplemented with 2%(v/v) B-27 minus insulin (Thermo Fisher Scientific), and from day 7 until the end of differentiation, cells were cultured in RPMI supplemented with 2%(v/v) B-27 (Thermo Fisher Scientific). At day 0 of differentiation, the Wnt signalling pathway was activated using the GSK3 inhibitor CHIR99021 (Stemgent) at a final concentration of 6 µM, in 2D conditions, and 3–18 µM, in 3D conditions. After 24 hours, medium was changed to RPMI + B27 minus insulin. At day 3, cells were cultured in basal medium supplemented with Wnt inhibitor IWP-4 (Stemgent) at a final concentration of 5 µM, for two days. At day 7, medium was changed and in the case of 3D culture, aggregates were transferred to Ultra-Low Attachment plates (Corning). Thereafter, medium was changed every 3 days until cell harvest.

### RNA sequencing and Data analysis

Total RNA from 2D monolayer and 3D aggregates at different stages of cardiac differentiation was extracted using High Pure RNA Isolation Kit (Roche), according to manufacturer´s instructions. RNA libraries were prepared for sequencing using Lexogen QuantSeq 3′mRNA-Seq Library Prep Kit FWD for Illumina using standard protocols. Sequencing was performed using Illumina HiSeq (50 cycle’s protocol) or NextSeq (75 cycle’s protocol) platforms. Sample read quality, reads mapping and counting were performed by a standard protocol from BlueBee Genomics Platform (http://www.bluebee.com/). With the RNA-Seq read counts matrix, we then used the DESeq2 (version 1.16.1) package of R to perform data normalization and differentially expressed genes (DEG) analysis. Information about DESeq2 package is available online at: https://bioconductor.org/packages/release/bioc/html/DESeq2.html. Gene ontology (GO) terms were identified using the PANTHER (protein annotation through evolutionary relationship) classification system (version 13.1)^60^. GO terms were identified by analysing differentially expressed genes using the following settings: GO Biological Process, test type FISHER, reference list *Homo Sapiens*. Heat maps and PCA using a selection of enriched genes were generated in the web tool ClustVis^61^ and in R.

### Statistical analysis

Statistical significance was determined using a Student’s t-test for all quantification except RNA-seq data. Data is represented as mean ± SEM for at least three replicate samples (see figure legends for additional information). Differential gene expression analysis via DESeq. 2 for RNA-seq data is described in the section “RNA-seq Data Analysis”.

### Accession numbers

RNA-seq data for this study are available through Gene Expression Omnibus (GEO) Accession Number GSE116574.

## Supplementary information

Supplementary Information

Table S1

Table S2

Table S3
