# Supplementary material for: Transcriptomic analysis of 3D Cardiac Differentiation of Human Induced Pluripotent Stem Cells Reveals Faster Cardiomyocyte Maturation Compared to 2D Culture
Source: Sci Rep. 2019 Jun 25;9:9229. doi: 10.1038/s41598-019-45047-9 (PMC6592905; doi:10.1038/s41598-019-45047-9)
Supplement: Supplementary file 1 — Supplementary Information [file 41598_2019_45047_MOESM1_ESM.pdf]

Transcriptomic analysis of 3D Cardiac Differentiation of Human Induced Pluripotent Stem Cells Reveals Faster Cardiomyocyte Maturation Compared to 2D Culture

Mariana A. Branco[1,2], João P. Cotovio[1,2], Carlos A.V. Rodrigues[1,2], Sandra H. Vaz[3,4], Tiago G. Fernandes[1,2], Leonilde M. Moreira[1], Joaquim M. S. Cabral[1,2], Maria Margarida Diogo[1,2*]

[1]Department of Bioengineering and iBB – Institute for Bioengineering and Biosciences, Instituto Superior Técnico, Universidade de Lisboa, 1049-001 Lisbon, Portugal

[2]The Discoveries Centre for Regenerative and Precision Medicine, Lisbon Campus, Instituto Superior Técnico, Universidade de Lisboa, 1049-001 Lisbon, Portugal

[3]Instituto de Medicina Molecular, Faculdade de Medicina, Universidade de Lisboa, 1649-028 Lisbon, Portugal

[4]Instituto de Farmacologia e Neurociências, Faculdade de Medicina da Universidade de Lisboa, 1649-028 Lisbon, Portugal

***** Correspondence: margarida.diogo@tecnico.ulisboa.pt

Supplementary Information

Supplementary Figures

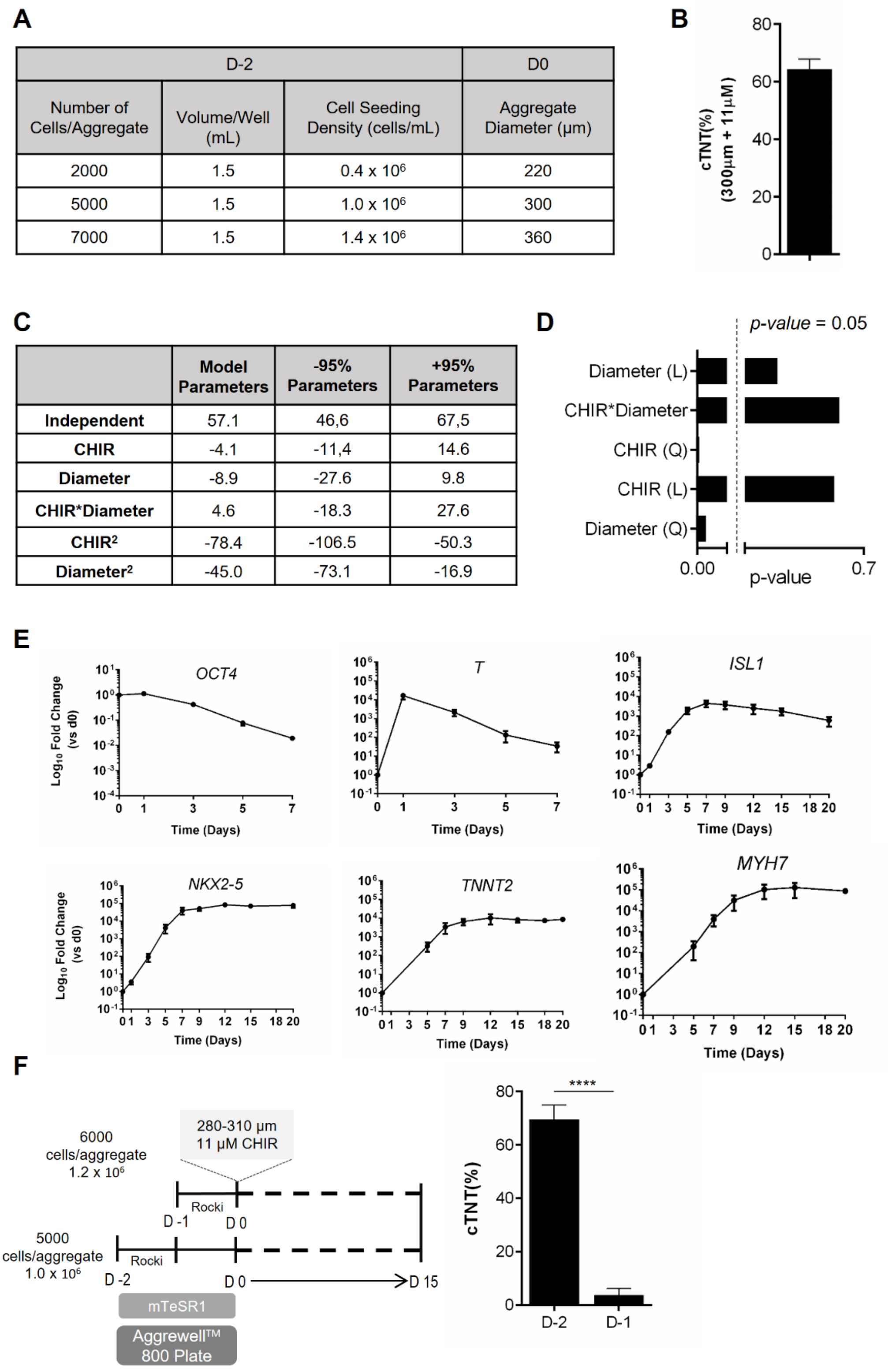

D-2			D0
Number of Cells/Aggregate	Volume/Well (mL)	Cell Seeding Density (cells/mL)	Aggregate Diameter (µm)
2000	1.5	0.4×10^6	220
5000	1.5	1.0×10^6	300
7000	1.5	1.4×10^6	360

	Model Parameters	-95% Parameters	+95% Parameters
**Independent**	57.1	46,6	67,5
**CHIR**	-4.1	-11,4	14.6
**Diameter**	-8.9	-27.6	9.8
**CHIR*Diameter**	4.6	-18.3	27.6
**CHIR²**	-78.4	-106.5	-50.3
**Diameter²**	-45.0	-73.1	-16.9

Figure S1. Optimization of hiPSC-3D cardiac differentiation using a forced aggregation platform. (**A)** Correspondence between cell seeding densities at day -2 (D-2) used in factorial design and aggregate diameters at day 0 (D0). (**B)** Percentage of $cTNT^+$ cells at D15 of cardiac differentiation for the centered point of the factorial design (aggregate diameter of 300µm and CHIR concentration of 11 µM). Data are represented as mean ± SEM, n=4 independent experiments. **(C)** Parameters of the quadratic model generated from the factorial design, which correlates the percentage of cTNT positive cells after 15 days of differentiation with CHIR concentration and aggregate diameter at D0. R^2=0.84. (**D)** Evaluation of the statistically significant contribution of each factor of the quadratic model. Only the quadratic terms of CHIR and aggregate diameter were statistically significant (p-values<0.05). (**E**) Expression profile of key genes during the time-course of 3D cardiac differentiation at the indicated time points, between day 0 and day 20, for a selected set of genes: pluripotency (*OCT4*), primitive streak (*T*), cardiac mesoderm (*ISL1* and *NKX2-5*) and cardiomyocyte (*TNNT2* and *MYH7*) markers. Values are normalized to *GAPDH* and relative to undifferentiated hiPSCs (day 0). Data are represented as mean ± SEM, n=4 independent experiments. (**F**) Percentage of $cTNT^+$ cells after 15 days of differentiation for both culture conditions for 1 day (D-1) and 2 days (D-2) of pre-differentiation period. Data are represented as mean ± SEM, n=6 independent experiments in the D-2 condition and n=3 independent experiments in the D-1 condition.

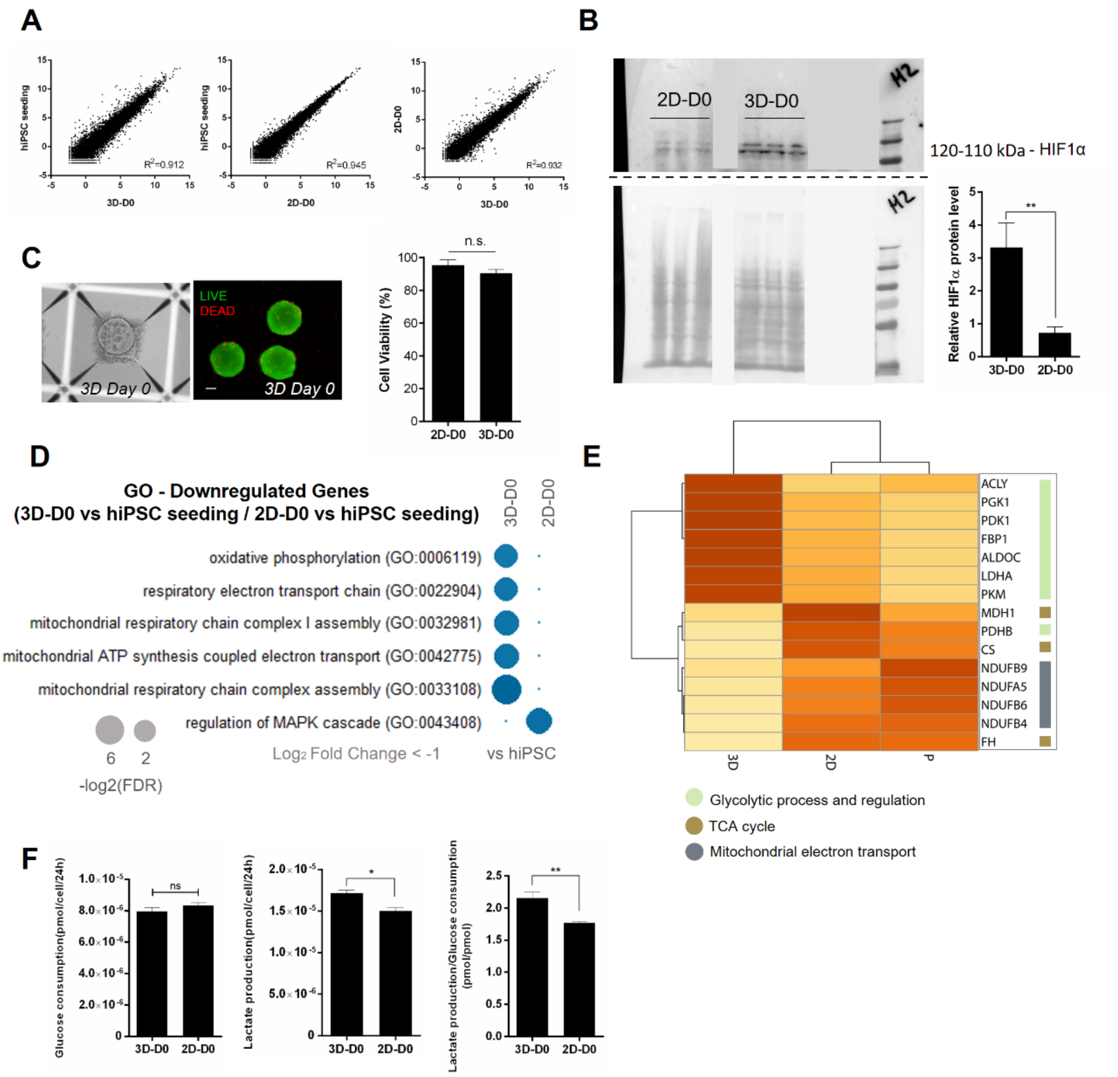

Figure S2. Impact of 3D culture of hiPSC during the pre-differentiation period before cardiac differentiation induction. (**A**) Correlation coefficients for 2D-D0 vs hiPSC seeding, 3D-D0 vs hiPSC seeding and 3D-D0 vs 2D-D0 show that D0 of both culture strategies are significantly correlated with hiPSC seeding population as expected, however in the 3D system there are already some genes that appear far from the linear correlation. Values used for the graph are Log_2 (CPM) for all RNA-seq data. (**B**) Western blot (left) and quantification (right) of HIF1α protein expression in 2D-D0 and 3D-D0 conditions. Data are represented as mean ± SEM, n=3 independent experiments. (**C**) Bright field and live/dead staining of aggregates at D0 of differentiation, showing high cell viability and no evidences of necrotic areas. Scale bar, 100 μm (left). Flow Cytometry analysis of viable cells at D0 of differentiation in both 2D and 3D culture conditions, proving that 3D culture do not compromise cell viability (right). Data are represented as mean ± SEM, n=3 independent experiments. (**D**) Gene ontology (GO) biological processes terms related with oxidative phosphorylation and mitochondrial respiratory chain identified (FDR < 0.05) for the differentially downregulated genes (Log2 FC < -1 and adjusted p-value < 0.05) of 3D-D0 versus hiPSC seeding and 2D-D0 versus hiPSC seeding. (**E**) Heat map highlighting differentially expressed genes in 3D-D0 versus hiPSC seeding populations (Log2 FC > 1 and adjusted p-value < 0.05) related with glycolysis metabolism and

TCA cycle. Average CPM values of 3 replicates were used for each condition. (**F**) Glucose consumption rate (pmol/cell/24hours), lactate production rate (pmol/cell/24hours) and yield of lactate produced/glucose consumed, at D0 of differentiation in both 3D aggregates and 2D monolayer. Data are represented as mean ± SEM, n=3 independent experiments. See Table S2 for full DE gene list.

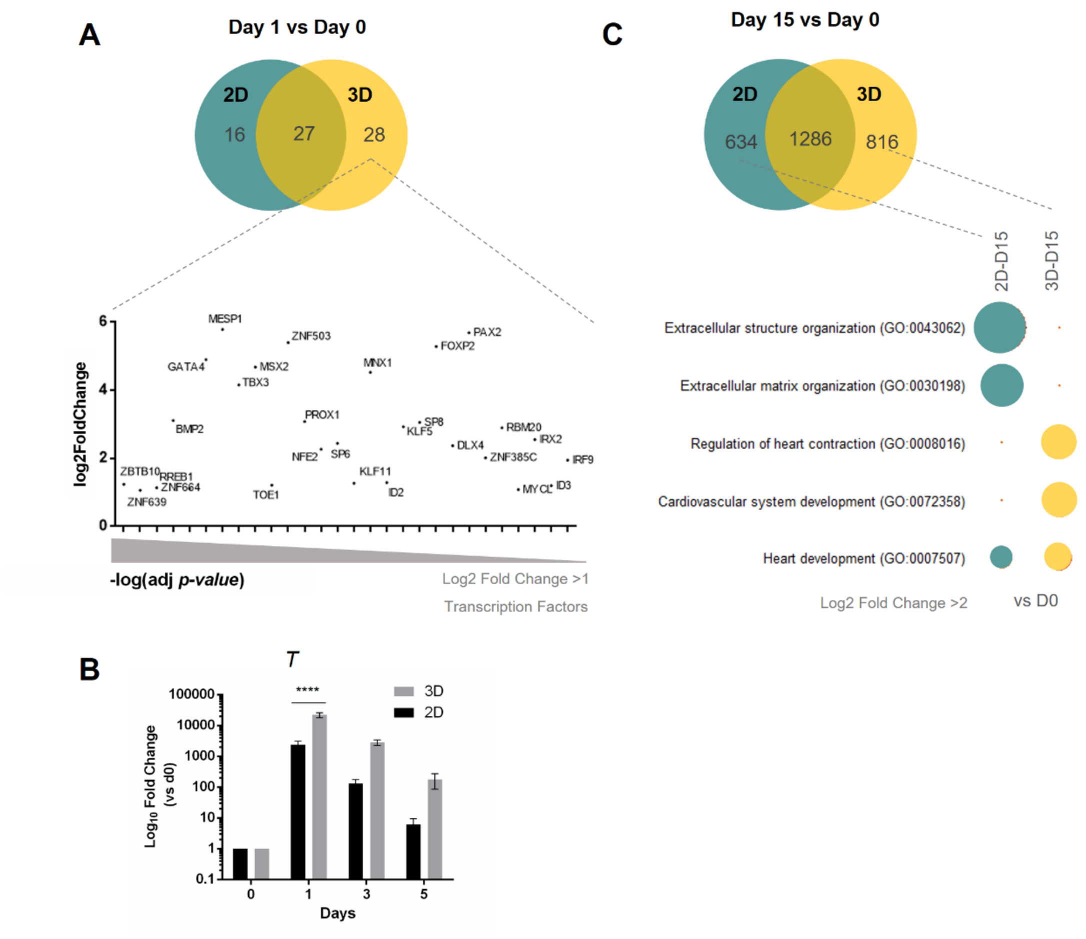

Figure S3: Differential expression analysis at Day 1 and Day 15 of cardiac differentiation in 3D cardiac differentiation compared to 2D monolayer culture system. (**A**) Venn diagram representing the number of transcription factors upregulated (Log2 FC > 1 and adjusted p-value < 0.05) in 3D-D1 and 2D-D1 versus 3D-D0 and 2D-D0, respectively, highlighting the ones that are only upregulated in 3D-D1 (bottom). (**B**) qRT-PCR analysis of the first 5 days of cardiac differentiation in both 3D and 2D, for mesendoderm gene T, highlighting the statistically significant higher expression of T after CHIR induction (day 1) in 3D aggregates compared with 2D monolayer. Values are normalized to GAPDH and relative to day 0. Data are represented as mean ± SEM, n=4 independent experiments. (**C**) Venn diagram (top) representing the number of genes (Log2 FC > 2 and adjusted p-value < 0.05) in 3D-D15 and 2D-D15 versus 3D-D0 and 2D-D0, respectively, and GO analysis (bottom) with the upregulated genes that are only present in 3D-D15 or 2D-D15.

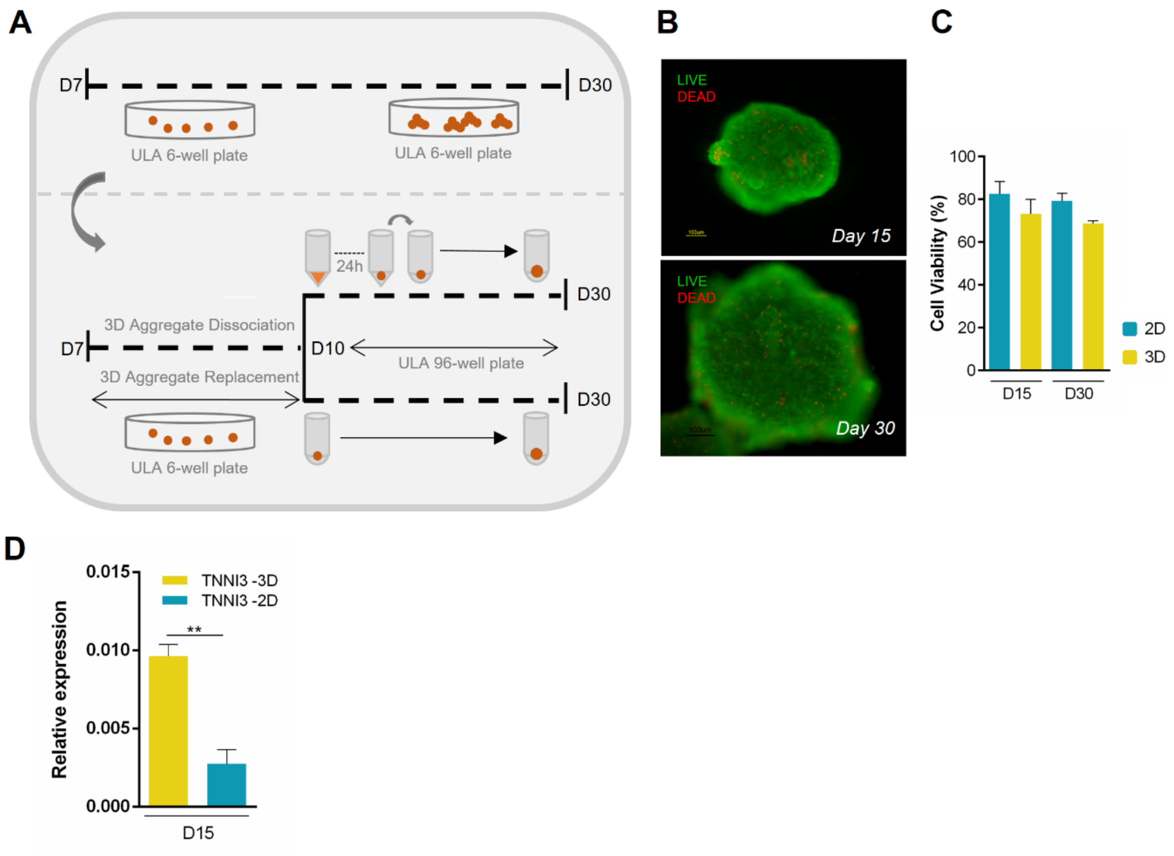

Figure S4. 3D cardiac differentiation platform optimization for long term culture of 3D cardiac aggregates. (**A**) Schematic representation of alternative methodologies for the long term maintenance in culture of 3D cardiac aggregates using 96-well plates. (**B**) live/dead staining of aggregates at D15 (top) and D30 (bottom) of differentiation, showing high cell viability. (**C**) Flow cytometry quantification of cell viability at D15 and D30 of differentiation for both culture conditions (2D and 3D). Data are represented as mean ± SEM, n=3 independent experiments. (D) Relative gene expression of cardiac troponin TNNI3 at D15 of differentiation in both culture conditions (2D and 3D). Values are normalized to *GAPDH* and *TNNT2*. Data are represented as mean ± SEM, n=3 independent experiments.

Supplementary Table Captions

Table S1. Related to Figures 2 and 3. RNA-seq data.

"CPM - Pluripotency" tab: Normalized gene expression (counts per million, CPM) for the initial hiPSC cell population, that was used for seeding both 2D and 3D culture formats ("hiPSC seeding"), and for the Day 0 samples for 2D and 3D culture conditions. For each culture condition and for each time point, triplicates were performed, for "hiPSC seeding samples", and are discriminated as "_1", "_2" and "_3".

"CPM - Differentiation 2D and 3D" tab: Normalized gene expression (counts per million, CPM) for samples collected from sequential stages of cardiac differentiation (Day 1, 3, 5, 7, 9, 12, 15, 18 and 20) for 2D and 3D cell culture conditions. For each culture condition and for each time point, triplicates were performed, and are discriminated as "_1", "_2" and "_3".

RNA-seq data for this study are available through Gene Expression Omnibus (GEO) accession number GSE116574

Table S2. Related to Figure 2. Differential gene expression analysis in the pre-differentiation period.

"DE gene list" tab: List of all differentially expressed (DE) genes with an adjusted p-value (padj) <0.05, for three different comparisons: "3D-D0 vs hiPSC seeding"; "2D-D0 vs hiPSCs seeding" and "3D-D0 vs 2D-D0". Differentially expressed gene analysis was performed using the package DESeq2 in R software

"GO - 3D D0 vs hiPSC seeding- up" and "GO - 3D-D0 vs 2D-D0 – up" tabs: Gene Ontology (GO) terms related with the most significant ($log_2FC > 2$) up regulated genes for both "3D D0 vs hiPSC seeding" and "3D-D0 vs 2D-D0" comparisons. The PANTHER classification system was used to identify the GO terms, considering only the ones with a false discovery rate (FDR) <0.05.

Table S3. Related to Figure 3. Differential gene expression analysis during cardiac differentiation.

"DE gene list" tab: List of all differentially expressed (DE) genes with an adjusted p-value (padj) <0.05, for three different comparisons: "3D-D5 vs 3D-D5"; "3D-D9 vs 3D-D9" and "3D-D20 vs 2D-D20". Differentially expressed gene analysis was performed using the package DESeq2 in R software.

"PCA 3D and 2D (D0 -D20)" tab: Principal component analysis (PCA) of RNA-seq data represented as average CPM (of triplicates) for a subset of 254 genes for each time point (D0, 1, 3, 5, 7, 9, 12, 15, 18, 20) and each type of culture (2D and 3D). Selected genes: cardiac differentiation process (mesoderm, cardiac mesoderm, and cardiac progenitor cells); cardiomyocytes (structure, contraction, cardiac action potential, Ca2+ handling, and metabolism); signaling pathways (WNT - canonical and non-canonical, NODAL/TGF-β/BMP, FGF); cardiomyocyte subtypes (ventricle/atrial/nodal specific genes).

"GO - D5 (3D vs 2D – up)", "GO – D9 (3D vs 2D – up)" and "GO- D20 (3D vs 2D – up)" tabs: Gene Ontology (GO) terms related with the most significant ($log_2FC > 1$, D5 and D9 and $log_2FC > 1.5$, D20) up

regulated genes for “3D-D5 vs 3D-D5”; “3D-D9 vs 3D-D9” and “3D-D20 vs 2D-D20” comparisons. The PANTHER classification system was used to identify the GO terms, considering only the ones with a false discovery rate (FDR) <0.05.

“HeatMap - D5 (3D vs 2D - up)”, “HeatMap – D9 (3D vs 2D - up)” and “HeatMap – D20 (3D vs 2D)” tabs: Heat map performed with log_2FC values for some of the upregulated or downregulated genes related with the most significant GO terms of “3D-D5 vs 3D-D5”; “3D-D9 vs 3D-D9” and “3D-D20 vs 2D-D20” comparisons.

Supplementary Experimental Procedures

Cell Maintenance

In this work, the optimization experiments were performed using the hiPSC line iPS-DF6-9-9T.B, provided by WiCell Bank. This cell line is vector free and was reprogramed from foreskin fibroblasts with a karyotype 46, XY that were collected from healthy donors using defined factors in the Laboratory of Dr. James Thomson, at University of Wisconsin. For the validation of the 3D differentiation platform, two additional hiPSC lines were used, hiPSC line F002.1A.13, derived from a healthy female donor using a standard protocol (Takahashi et al., 2007) provided by TCLab (Tecnologias Celulares para Aplicação Médica, Unipessoal, Lda.) and Gibco® Human Episomal iPSC line (iPSC6.2) (Burridge et al., 2011). The hiPSCs were maintained in mTeSR™1 (StemCell Technologies) in six-well plates coated with Matrigel™ (Corning). Medium was changed daily. Cells were routinely passaged every three to four days using 0.5mM EDTA solution (Thermo Fisher Scientific).

Cardiomyocyte hiPSC differentiation in 2D and 3D culture conditions

Pre-differentiation period. For 2D monolayer culture, cells were seeded onto Matrigel coated 12-well tissue culture plates at a cell density of $4x10^5$ cells/well. Culture medium was changed daily until a confluence of around 90-95%. For 3D aggregates formation, cells were incubated with ROCK inhibitor (ROCKi, Y-27632, 10µM, StemCell Technologies) at 37ºC for 1 h and then treated with accutase (Sigma) for 7 min at 37ºC. After dissociation, cells were quickly re-aggregated using microwell plates (AggreWell™800, StemCell Technologies) according to the manufacturer's instructions. Cells were plated at different densities in 1.5 mL/well of mTeSR™1 supplemented with 10µM ROCKi. 24 hours later, total volume of medium was replaced and cells were maintained in mTeSR™1 without ROCKi for an additional two days. ***hiPSCs differentiation.*** For hiPSCs differentiation into cardiomyocytes, in both culture conditions, an adapted GiWi protocol was used (Lian et al., 2013). RPMI 1640 medium (Thermo Fisher Scientific) was used as basal medium. From day 0 to day 7, cells were cultured in RPMI supplemented with 2%(v/v) B-27 minus insulin (Thermo Fisher Scientific), and from day 7 until the end of differentiation, cells were cultured in RPMI supplemented with 2%(v/v) B-27 (Thermo Fisher Scientific). At day 0 of differentiation, the Wnt signaling pathway was activated using the GSK3 inhibitor CHIR99021 (Stemgent) at a final concentration of 6 µM in 2D and 11 µM in 3D culture conditions. After 24 hours, medium was changed. At day 3, cells were cultured in basal medium supplemented with Wnt inhibitor IWP-4 (Stemgent) at a final concentration of 5 µM, for two days. At day 7, medium was changed and in the case of 3D culture, aggregates were flushed from the AggreWell™800 plate and transferred to 6-Well Ultra-Low Attachment plates. Thereafter, medium was changed every 3 days until cell harvest. ***Aggregate size monitoring.*** To monitor aggregate sizes throughout time in culture, several images were acquired at different time points using a Leica DMI 3000B microscope with a Nikon DXM 1200F digital camera. Aggregates were measured using ImageJ Software.

Expression profiling with RNA sequencing

Sample collection and RNA extraction. Samples from 2D monolayer and 3D aggregates at different stages of cardiac differentiation were singularized with 0.25% trypsin-EDTA at 37ºC for (7-15) min. For enzymatic digestion neutralization, RPMI+10% FBS was added. After centrifugation and washing cells with PBS, the cell pellet was frozen at -80ºC. Total RNA from the samples was extracted using High Pure RNA Isolation Kit (Roche, Cat. 11828665001), according to manufacturer´s instructions. ***RNA-seq sample preparation and sequencing.*** RNA libraries were prepared for sequencing using Lexogen QuantSeq 3'mRNA-Seq Library Prep Kit FWD for Illumina using standard protocols. Briefly, 500 ng of total RNA were primed with the oligo dT primer containing Illumina-compatible linker sequences. After first strand synthesis, the RNA was removed and second strand synthesized with Illumina-compatible random primers. After magnetic bead-based purification, the libraries were PCR amplified introducing the sequences required for cluster generation. Sequencing was performed using HiSeq (50 cycles protocol) or NextSeq (75 cycles protocol) platforms. Sequencer Software HiSeq Control Software 2.2.58 was used for base calling of samples processed on Illumina HiSeq 2000. Base calling of samples processed in NextSeq Sequencer was performed with the Real-Time Analysis (RTA) v2. ***RNA-seq Data Analysis.*** Sample read quality, reads mapping and counting were performed by a standard protocol from BlueBee Genomics Platform (http://www.bluebee.com/). ***Differential Gene Expression Analysis.*** With the RNA-Seq read counts matrix, we then used the DESeq2 (version 1.16.1) package of R to perform data normalization and differentially expressed genes (DEG) analysis. Information about DESeq2 package is available online at https://bioconductor.org/packages/release/bioc/html/DESeq2.html. ***Gene Ontology analysis and data representation.*** Gene ontology (GO) terms were identified using the PANTHER (protein annotation through evolutionary relationship) classification system (version 13.1) (Mi, Muruganujan, Casagrande, & Thomas, 2013). GO terms were identified by analyzing differentially expressed genes using the following settings: GO Biological Process, test type FISHER, reference list Homo Sapiens and FDR<0.05. Heat maps and PCA using a selection of enriched genes were generated in the web tool ClustVis (Metsalu & Vilo, 2015) and in R.

Flow Cytometry

Sample collection. For flow cytometry analysis, cells were washed with PBS and then incubated with Accutase in the case of hiPSC, or singularized with 0.25% trypsin-EDTA, in the case of differentiating hiPSC, at 37ºC for (7-15) min. For enzymatic digestion neutralization, FBS-containing medium was added. After centrifugation and washing the cell pellet, cells were fixed with 2% paraformaldehyde (PFA) reagent for 20 minutes at RT or stored at 4ºC. ***hiPSC (Intracellular and Surface markers).*** For intracellular staining, samples were centrifuged at 1000 rpm for 5 minutes and washed twice with 1% normal goat serum (NGS) solution in PBS. Then, cells were incubated in 1:1 of 3% NGS and 1% saponin (Sigma) in PBS, at room temperature for 15 min, for cell membrane permeabilization. After washing 3 times with 1% NGS, primary antibody was diluted in 3% NGS, and cells incubated at RT for 90 min. After incubation, cells were then washed with 1% NGS, and after centrifugation, cell pellet was resuspended and incubated with secondary antibody diluted in 1% NGS, at room temperature for 45 min in the dark. Cells were washed twice with 1% NGS, centrifuged and resuspended in PBS for a final volume of 300 μL/FACS tube. For

staining cell surface markers, cells were washed twice with PBS and re-suspended in primary antibody (Table S4) diluted in FACS buffer, at approximately 500,000 cells per condition, and incubated for 30 min at RT. Afterwards, cells were washed with PBS and re-suspended in secondary antibody diluted in FACS buffer for another 15 min, at room temperature (RT) in the dark. In the case of conjugated antibodies a single incubation period of 20 minutes at RT and in the dark was performed. Finally, cells were washed twice with PBS for a final volume of 300 µL/FACS tube. ***Differentiated hiPSC-derived cells***. Samples previously stored in 2% PFA, were centrifuge at 1000 rpm for 3 minutes and then incubated with 90% (v/v) cold methanol at 4ºC for 15 min. Cells were then washed 3 times with flow cytometry buffer 1 (FB1), constituted by 0.5% bovine serum albumin (BSA) solution in PBS. Cell pellet was resuspended and incubated with the primary antibody diluted in FB2, constituted by 0.1% Triton X-100 in FB1, at room temperature for 1 h. After incubation, cells were washed with FB2 and cell pellet was resuspended and incubated in the secondary antibody diluted in FB2, at room temperature for 30 min in the dark. Cells were washed twice with FB2. Cell pellet were resuspended in FB1 for a final volume of 300 µL/FACS tube. ***Data acquisition and analysis***. Flow cytometry was performed using a FACSCalibur™ flow cytometer (by Becton Dickinson) and data analysis was performed using Flowing Software 2.0.

Live/Dead assay

Cells were washed with PBS and then incubated with Accutase, in the case of hiPSC, or singularized with 0.25% trypsin-EDTA, in the case of differentiating hiPSC, at 37ºC for (7-15) min. For enzymatic digestion neutralization, FBS-containing medium was added. After centrifugation and washing the cell pellet, cells were incubated with LIVE/DEAD™ Fixable Dead Cell Stain Kit (invitrogen) for 15 min. After that period, cells were washed with PBS, resuspended in PBS for a final volume of 300 µL/FACS tube and run in flow cytometer for cell viability data acquisition.

Total protein extraction and Immunoblotting

hiPSCs were collected and lysed using ice-cold lysis buffer (10 mM Tris-HCl, pH 7.6, 5 mM MgCl2, 1.5 mM potassium acetate, 1% Nonidet P-40, 2 mM DTT) and 1X Halt Protease and Phosphatase Inhibitor Cocktail (Thermo Fisher Scientific, Inc.) for 30 min at 4 °C. Samples were then sonicated for 30 sec in ultrasound and then centrifuge for 10 min at 10,000g and 4 °C, saving supernatant. Total protein content was measured using the BioRad protein assay kit (Bio-Rad Laboratories, Hercules, CA, USA), according to the manufacturer’s specifications. Protein extracts were then denatured with 6x loading buffer. Equivalent amounts of protein (100 µg) were separated in 8% SDS-PAGE gel electrophoresis and then transferred onto nitrocellulose membrane. The membranes were further blocked with 5% milk in TBS: 25 mM Tris-HCl, 150 mM NaCl, pH 7.6), during 1 h at room temperature and further incubated overnight at 4°C with gentle agitation with primary antibody HIF1a (ReD Systems, MAB1536) prepared in 5% milk in 1xTBS. Membranes were incubated with anti-mouse IgG secondary antibody conjugated with horseradish peroxidase (1:5000, Bio-Rad Laboratories) for 2h at RT. Membranes were processed for protein detection using SuperSignal substrate (Pierce, Thermo Fisher Scientific). Finally, the relative intensities of protein bands were analyzed using ImageLab Version 5.1 densitometric analysis program (Bio-Rad Laboratories).

In order to normalize the amount of protein per lane, Ponceau staining was used. Protein Ladder, 10 to 250 kDa (catalog number, 26619) was used.

Glucose consumption and Lactate production rate analysis

To determine glucose consumption rate and lactate production rate, mTeSR™1 exhausted medium from 2D-D0 and 3D-D0 was collected. Glucose and lactate concentrations from cell-free supernatants were analyzed using an YSI 2700 Select™ Biochemistry Analyzer (YSI Incorporated Life Sciences, USA). Fresh mTeSR™1 was used to determine the concentration of glucose initially present in the culture media. The yield of lactate from glucose was calculated for each day as ΔLac/ΔGlc, where ΔLac is the production of lactate during that day and ΔGlc is the consumption of glucose during the same period.

Immunostaining

Sample collection. Samples from 2D culture or 3D aggregates were fixed in 4% paraformaldehyde (PFA, Sigma) at 4ºC for 30 min. After PFA removal, cells were stored in Phosphate buffered saline (PBS, 0.1M) at 4ºC for further analysis. 3D aggregates were incubated in 15% (m/v) sucrose in PBS, at 4ºC overnight and afterwards embedded in 7.5%/15% gelatin/sucrose and frozen in isopenthane at -80ºC. Aggregates with twelve-μm sections were cut on a cryostat-microtome (Leica CM3050S, Leica Microsystems), collected on Superfrost™ Microscope Slides (Thermo Scientific) and stored at -20ºC. Sections were de-gelatinized for 45 min in PBS at 37ºC before immunohistochemistry. ***Staining.*** 3D aggregate sections and replated cells in coverslips were incubated in 0.1 M Glycine (Millipore) for 10 min at room temperature to remove PFA residues, permeabilized with 0.1% Triton X-100 (Sigma), at room temperature for 10 min and blocked with 10% fetal goat serum (FGS, Gibco) in TBST (20 mM Tris-HCl pH 8.0, 150 mM NaCl and 0.05% Tween-20, Sigma), at room temperature for 30 min. Cells were then incubated with the primary antibody diluted in blocking solution (Table S4) at 4ºC overnight. Secondary antibodies were added for 30 min and nuclear counterstaining was performed using 4',6-diamidino-2-phenylindole (DAPI, 1.5μg/mL, Sigma), at room temperature for 5 min. After brief drying, sections were mounted in Mowiol (Sigma). ***Image acquisition and analysis*** Immunofluorescence staining images were acquired with a LSM 710 Confocal Laser Point-Scanning Microscope (by Zeiss) for 3D aggregate sections and replated samples; data analysis was performed using ZEN Imaging Software (by Zeiss) and ImageJ Software.

Table S4 List of primary and secondary antibodies used in Flow Cytometry and Immunostaining

Antibody	Source	Reference	Isotype	Dilution
CD31	Dako	M0823	Mouse IgG	1:100 (IS) 1:40 (FC)
cTNT	Thermo Fisher	MA5-12960	Mouse IgG	1:200 (IS) 1:800 (FC)
α-SMA	Sigma-Aldrish	161208D	Rabbit IgG	1:200 (IS/FC)
CD90-PE	Biolegend	328110	Mouse IgG	1:6 (FC)
CX43	Sigma-Aldrish	C6219	Rabbit IgG	1:400 (IS)
OCT4	Millipore	288465	Mouse IgG	1:200 (IS/FC)
Calponin	Abcam	ab700	Mouse IgG1	1:200 (FC)
Ki-67	Abcam	ab833	Rabbit IgG	1:150 (IS)

TRA-1-60 - PE	Miltenyi Biotec	130-100-347	Human IgG	1:11 (FC)
SSEA-4 - PE	Miltenyi Biotec	130-098-369	Human IgG	1:11 (FC)
Secondary Antibody	Thermo Fisher	A11001	Alexa 488 Goat anti-Mouse IgG	1:500 (IS) 1:1000 (FC)
Secondary Antibody	Thermo Fisher	A11008	Alexa 488 Goat anti-Rabbit IgG	1:500 (IS) 1:1000 (FC)
Secondary Antibody	Thermo Fisher	A11003	Alexa 546 Goat anti-Mouse IgG	1:500 (IS)
Secondary Antibody	Thermo Fisher	A11010	Alexa 546 Goat anti-Rabbit IgG	1:500 (IS)

Quantitative real time (qRT)-PCR

Total RNA from cell samples of sequential stages of cardiomyocyte differentiation was extracted using High Pure RNA Isolation Kit (Roche) according to manufacturer´s instructions, and converted into cDNA with High Capacity cDNA Reverse Transcription Kit (Thermo Fisher Scientific). PCR reactions were performed with Taqman™ Gene Expression Assays (Thermo Fisher Scientific) or SYBR Green Master Mix (nzytech) (Table S5). Reactions were run in triplicate in ViiA7 Real-Time PCR Systems (Applied BioSystems). For each analysed time point, gene expression was normalized against the expression of the housekeeping gene glyceraldehyde-3-phosohate dehydrogenase (*GAPDH*) and results analyzed with QuantStudio^{TM} RT-PCR Software.

Table S5 List of primers used in qRT-PCR

Gene	Assay ID
GAPDH	Hs02758991_g1
OCT4	Hs00999634_gH
T	Hs00610080_m1
ISL1	Hs01099687_m1
NKX2-5	Hs00231763_m1
TNNT2	Hs00165960_m1
TNNI3	Hs00165957_m1

Gene	Sequence
FOXA2	FW - GGGAGCGGTGAAGATGGA<br>RV - TCATGTTGCTCACGGAGGAGTA
LEFTY1	FW - GAAGTGTTCGAGGGTACCAGG<br>RV - AAAACTGAGCAAGGGCTCTCC
NODAL	FW – CCCAAGCAGTACAACGCCTA<br>RV – TGCATGGTTGGTCGGATGAA
CER1	FW - TTCTCAGGGGGTCATCTTGC<br>RV - ATGAACAGACCCGCATTTCC
JUN	FW - TTCTATGACGATGCCCTCAACGC<br>RV - GCTCTGTTTCAGGATCTTGGGGTTAC
FOS	FW - GCATCTGCAGCGAGCATCTGAGAA<br>RV – AGAGCTGGGTAGGAGCACGGTCACT
PGK1	FW - CAAGAAGTATGCTGAGGCTGTCA<br>RV - CAAATACCCCCACAGGACCAT
BNIP3	FW - CAGGGCTCCTGGGTAGAACT

	RV - CTCCGTCCAGACTCATGCTG
GDF3	FW - CATGCCGTTGACCCAGAGAT<br>RV - ACCCACACCCACATTCATCG

Patch-clamp recordings

Whole cell patch-clamp recordings were obtained from CMs dissociated from aggregates at day 30 of differentiation using an upright microscope (Zeiss Axioskop 2FS) equipped with differential interference contrast optics using a Zeiss AxioCam MRm camera and a x40 IR-Achroplan objective. Spontaneous Action Potentials (Aps) from CMs dissociated from aggregates were recorded in the current-clamp mode using a Axopatch 200B (Axon Instruments) amplifier. The patch pipette (4- to 7-M resistance) was filled with an internal solution containing (in mM): 125 K-gluconate, 11 KCl, 0.1 CaCl2, 2 MgCl2, 1 EGTA, 10 HEPES, 2 MgATP, 0.3 NaGTP, and 10 phosphocreatine, pH 7.3, adjusted with 1 M NaOH, 280 –290 mosM. The bath recording solution consisted of (in mM): 124 NaCl, 3 KCl, 1.25 NaH_2PO_4, 26 $NaHCO_3$, 1 $MgSO_4$, 2 $CaCl_2$, and 10 glucose, gassed with 95% O2-5% CO2, pH 7.4. The recordings were performed at 37 °C and offset potentials were nulled before gigaseal formation. Aps were recorded right after establishing whole-cell configuration. Acquired signals were filtered using an in-built, 2-kHz, 3-pole Bessel filter, and data were digitized at 5 or 10 kHz under control of the pCLAMP 10 (Molecular Devices) software program. We analyzed AP duration at 50% and 90% repolarization (APD_{50} and APD_{90}, respectively). Parameters from 5 consecutive APs were averaged.

Supplementary References

Burridge, P. W., Matsa, E., Shukla, P., Lin, Z. C., Churko, J. M., Ebert, A. D., … Wu, J. C. (2014). Chemically defined generation of human cardiomyocytes. *Nature Methods*, *11*(8), 855–860. https://doi.org/10.1038/nmeth.2999

Burridge, P. W., Thompson, S., Millrod, M. A., Weinberg, S., Yuan, X., Peters, A., … Zambidis, E. T. (2011). A universal system for highly efficient cardiac differentiation of human induced pluripotent stem cells that eliminates interline variability. *PLoS ONE*, *6*(4). https://doi.org/10.1371/journal.pone.0018293

Lian, X., Zhang, J., Azarin, S. M., Zhu, K., Hazeltine, L. B., Bao, X., … Palecek, S. P. (2013). Directed cardiomyocyte differentiation from human pluripotent stem cells by modulating Wnt/β-catenin signaling under fully defined conditions. *Nature Protocols*, *8*, 162–75. https://doi.org/10.1038/nprot.2012.150

Metsalu, T., & Vilo, J. (2015). ClustVis: A web tool for visualizing clustering of multivariate data using Principal Component Analysis and heatmap. *Nucleic Acids Research*, *43*(W1), W566–W570. https://doi.org/10.1093/nar/gkv468

Mi, H., Muruganujan, A., Casagrande, J. T., & Thomas, P. D. (2013). Large-scale gene function analysis with the panther classification system. *Nature Protocols*, *8*(8), 1551–1566. https://doi.org/10.1038/nprot.2013.092

Takahashi, K., Tanabe, K., Ohnuki, M., Narita, M., Ichisaka, T., Tomoda, K., & Yamanaka, S. (2007). Induction of pluripotent stem cells from adult human fibroblasts by defined factors. *Cell*, *131*(5), 861–72. https://doi.org/10.1016/j.cell.2007.11.019
